# Identification of Siglec-9 as the receptor for MUC16 on human NK cells, B cells, and monocytes

**DOI:** 10.1186/1476-4598-9-118

**Published:** 2010-05-24

**Authors:** Jennifer A Belisle, Sachi Horibata, Gubbels AA Jennifer, Sarah Petrie, Arvinder Kapur, Sabine André, Hans-Joachim Gabius, Claudine Rancourt, Joseph Connor, James C Paulson, Manish S Patankar

**Affiliations:** 1 Department of Obstetrics and Gynecology, University of Wisconsin-Madison, Madison, WI; USA; 2 Institute for Physiological Chemistry, Faculty of Veterinary Medicine, Ludwig-Maximilians-University, Munich, Germany; 3 Department of Microbiology and Infectiology, Universite de Sherbrooke, Sherbrooke, Canada; 4 The Scripps Research Institute, Departments of Chemical Physiology and Molecular Biology, La Jolla, CA, USA

## Abstract

**Background:**

MUC16 is a cell surface mucin expressed at high levels by epithelial ovarian tumors. Following proteolytic cleavage, cell surface MUC16 (csMUC16) is shed in the extracellular milieu and is detected in the serum of cancer patients as the tumor marker CA125. csMUC16 acts as an adhesion molecule and facilitates peritoneal metastasis of ovarian tumors. Both sMUC16 and csMUC16 also protect cancer cells from cytotoxic responses of natural killer (NK) cells. In a previous study we demonstrated that sMUC16 binds to specific subset of NK cells. Here, we identify the csMUC16/sMUC16 binding partner expressed on immune cells.

**Results:**

Analysis of immune cells from the peripheral blood and peritoneal fluid of ovarian cancer patients indicates that in addition to NK cells, sMUC16 also binds to B cells and monocytes isolated from the peripheral blood and peritoneal fluid. I-type lectin, Siglec-9, is identified as the sMUC16 receptor on these immune cells. Siglec-9 is expressed on approximately 30-40% of CD16^pos^/CD56^dim ^NK cells, 20-30% of B cells and >95% of monocytes. sMUC16 binds to the majority of the Siglec-9^pos ^NK cells, B cells and monocytes. sMUC16 is released from the immune cells following neuraminidase treatment. Siglec-9 transfected Jurkat cells and monocytes isolated from healthy donors bind to ovarian tumor cells via Siglec-9-csMUC16 interaction.

**Conclusions:**

Recent studies indicate that csMUC16 can act as an anti-adhesive agent that blocks tumor-immune cell interactions. Our results demonstrate that similar to other mucins, csMUC16 can also facilitate cell adhesion by interacting with a suitable binding partner such as mesothelin or Siglec-9. Siglec-9 is an inhibitory receptor that attenuates T cell and NK cell function. sMUC16/csMUC16-Siglec-9 binding likely mediates inhibition of anti-tumor immune responses.

## Introduction

MUC16 is a membrane spanning mucin that is expressed on ovarian, endometrial, tracheal, and ocular surface epithelial cells [1-4]. This mucin is initially expressed on the surface (cell surface MUC16, csMUC16) and then shed (shed MUC16, sMUC16) in the extracellular milieu following proteolytic cleavage [5-7]. csMUC16 carries a ~12,000 amino acid N-terminal region, a Variable Number of Tandem Repeat (VNTR) domain composed of 60 tandem repeats of 156 amino acids, and a 256 amino acid cytoplasmic tail [7-9]. The mucin is heavily glycosylated with both O- and N-linked oligosaccharides [10]. Because of all of these structural features, the average molecular weight of csMUC16 and sMUC16 is between 3-5 million Da [7,9,10].

csMUC16 is overexpressed by human epithelial ovarian tumor cells [11,12]. sMUC16 is released by ovarian tumors and can be detected in the peritoneal fluid and peripheral blood of cancer patients. A repeating peptide epitope present in the VNTR domain of csMUC16 and sMUC16 has been previously identified as the tumor marker CA125. Elevations, from an individualized nadir serum concentration of CA125, are routinely determined in order to monitor progression of epithelial ovarian cancer in patients undergoing treatment for this disease [13,14].

In addition to serving as a cancer biomarker, MUC16 is also important in promoting the metastasis and growth of ovarian tumors. csMUC16 facilitates tumor cell aggregation and their subsequent binding to the peritoneal surfaces by serving as a ligand of mesothelin, a glycoprotein expressed on mesothelial cells [15-17]. Both csMUC16 and sMUC16 protect ovarian tumor cells from immune attack and thereby promote tumor growth. sMUC16 is an inhibitor of the cytolytic anti-tumor responses of natural killer (NK) cells [18]. On the other hand, csMUC16 acts as an anti-adhesive molecule and prevents the formation of the immunological synapse between ovarian cancer cells and NK cells [19]. Additional studies are required to carefully delineate the immunoprotective role of sMUC16 and csMUC16 and their contribution to the progression of ovarian tumorigenesis.

Phenotypic analysis of NK cells isolated from the peripheral blood and peritoneal fluid of ovarian cancer demonstrated that sMUC16 derived from the tumors binds strongly to the surface of a select subset of CD16^pos^/CD56^dim ^NK cells [20]. RT-PCR of peripheral blood mononuclear cells (PBMC) from ovarian cancer patients and other in vitro experiments, reported in our previous study, showed that immune cells do not express MUC16 [20]. sMUC16 was detected on the immune cell surface using the murine monoclonal antibodies VK8, and OC125 [20]. Both VK8 and OC125 are highly specific anti-MUC16 antibodies. VK-8 was used in experiments that ultimately led to the cloning of MUC16 [6,21] and the OC125 antibody was used as a reagent to establish the clinical-grade CA125 assay to measure the concentration of this marker in the sera of cancer patients [22,23]. A third antibody, 618F, developed by our group [24], also binds specifically to subsets of CD16^pos^/CD56^dim ^NK cells isolated from ovarian cancer patients but not to those obtained from healthy donors (Patankar et al unpublished data). Lack of endogenous MUC16 expression and the binding of highly specific anti-MUC16 antibodies to only fixed subsets of patient derived immune cells suggested the presence of a MUC16 receptor on immune cells. The current study was therefore undertaken to identify the binding partner for MUC16 on immune cells.

Here, we report that sMUC16 selectively binds not only to a subset of CD16^pos^/CD56^dim ^NK cells but also is present on the surface of approximately 20% of the CD19^pos ^B cells and >90% of all CD14^pos ^monocytes. Additional experiments demonstrate that both sMUC16 and csMUC16 bind to the immune cell surface via the I-type lectin Siglec-9. csMUC16-Siglec-9 binding mediates adhesion of immune cells with ovarian tumor cells. Identification of Siglec-9 as the sMUC16/csMUC16 binding partner provides an opportunity to identify the molecular mechanisms that lead to suppression of immune responses by this mucin. Apart from its importance in understanding the biological role of MUC16, the data also suggests that sMUC16 binding to immune cells may serve as a novel strategy for the detection and monitoring of ovarian cancer.

## Results

### Potential sMUC16 receptors on CD16^pos^/CD56^dim ^NK cell surface

Human NK cells are classified into two major phenotypes, CD16^pos^/CD56^dim ^and CD16^neg^/CD56^bright^. The CD16^pos^/CD56^dim ^NK cells are highly cytotoxic NK cells whereas the CD16^neg^/CD56^bright ^NK cells are not efficient in lysing tumor targets [25,26]. Because sMUC16 binds only to subsets of CD16^pos^/CD56^dim ^NK cells and also inhibits their cytolytic activity, we postulated that sMUC16 is likely a ligand of an inhibitory receptor on NK cells. Since sMUC16 is heavily glycosylated, we specifically considered carbohydrate binding receptors of NK cells as potential binding partners.

The lectin galectin-1 has been reported as a MUC16 receptor on NK cells [27,28]. However, galectin-1 is primarily expressed by CD16^dim^/CD56^bright ^NK cells (Additional file 1) while MUC16 is bound by CD16^pos^/CD56^dim ^NK cells. Therefore, galectin-1 was considered unlikely to be involved in mediating binding of sMUC16 to CD16^pos^/CD56^dim ^NK cells. Another binding partner for sMUC16 is mesothelin [15]. This glycoprotein is however not expressed on NK cells based on microarray analysis [29].

**Figure 1:**
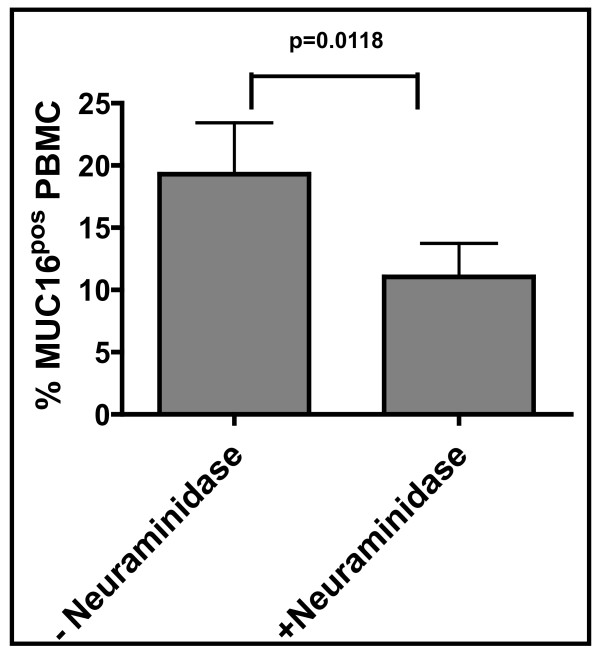
**sMUC16 binding to immune cells is reduced following neuraminidase treatment**. PBMC from each of six ovarian cancer patients were digested with neuraminidase from *Clostridium perfringens *in phosphate buffered saline (PBS). Matching control PBMC from each of the six patients were treated with heat inactivated neuraminidase (*Clostridium perfringens*). sMUC16 binding to the control and enzymatically digested cells was measured by flow cytometry using the anti-MUC16 antibody, VK-8. Each bar is the mean measurement for all six donors. Data was analyzed using the Wilcoxon signed rank test.

To test if sMUC16 was binding to immune cells via its oligosaccharides, we digested the peripheral blood mononuclear cells (PBMC) from ovarian cancer patients with neuraminidase from *Clostridium perfringens*. After a limited (15 min) digestion with neuraminidase, the PBMC were monitored by flow cytometry for the amount of sMUC16 still bound to the cell surface. Neuraminidase treatment resulted in a loss of sMUC16 binding on approximately 50% of the PBMCs as compared to matching controls not treated with neuraminidase (Figure 1).

NK cells are reported to express two members of the sialic acid binding Siglec family, Siglec-7 and Siglec-9 [30,31]. Siglec-9 recognizes α2-3-linked sialic acid where as Siglec-7 preferentially binds to glycans terminated with α2-8-linked sialic acid residues [32,33]. Glycomic analysis has revealed extensive expression of α2-3-linked sialic acid residues on MUC16 [10]. Therefore, we focused our attention on Siglec-9 as a potential candidate immune cell receptor for this mucin.

### sMUC16 predominantly binds to Siglec-9^pos^/CD16^pos^/CD56^dim ^NK cells

**Figure 2:**
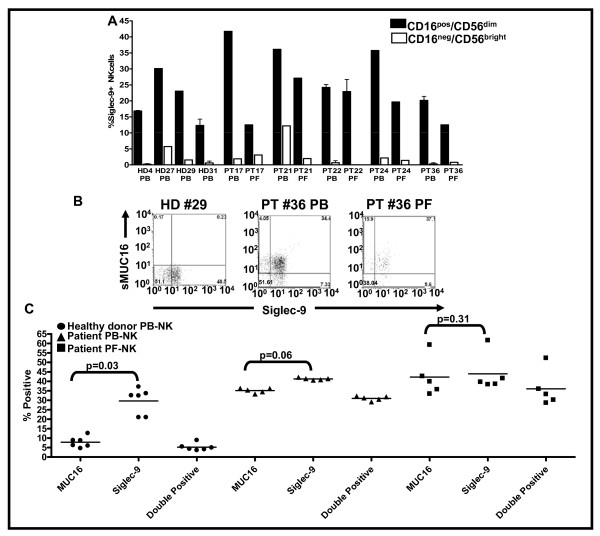
**sMUC16 predominantly binds to Siglec-9^pos^/CD16^pos^/CD56^dim ^NK cells**. Peripheral blood mononuclear cells (PBMC) from healthy donors (HD) and patients (PT) and peritoneal fluid mononuclear cells (PFMC) of PT were labeled with VK8 followed by a fluorophore conjugated secondary reporter antibody. The cells were subsequently labeled with fluorescently tagged anti-CD3, -CD16, -CD56, -Siglec-9, and -NKp46 antibodies as described earlier [20]. NK cells identified as CD3^neg^/NKp46^pos ^cells were classified into CD16^pos^/CD56^dim ^and CD16^neg^/CD56^bright ^cells by flow cytometry. ***A***, Siglec-9 expression on the two NK cell subpopulations, CD16^pos^/CD56^dim ^and CD16^neg^/CD56^bright^, was analyzed. Each bar represents a mean and standard deviation of three independent experiments. ***B and C***, sMUC16 binding and Siglec-9 expression on CD16^pos^/CD56^dim ^NK isolated from PBMC of healthy donors and PBMC and PFMC of cancer patients was determined. **B**, shows dot plots of representative healthy donor (HD) peripheral blood NK and patient (PT) NK cells from peripheral blood (PB) and peritoneal fluid (PF). Majority of events in the lower left quadrant are located on the axes and therefore not visible in the dot plots. ***C***, Each value is mean of PBMC from 6 healthy donor and PBMC and PFMC from five cancer patients showing percent of the cells positive for Siglec-9 and sMUC16 and those double positive for both Siglec-9 and sMUC16 are shown.

Analysis of NK cells from the peripheral blood (PB) of four healthy donors indicated that Siglec-9 was detectable only on approximately 15-40% of the CD16^pos^/CD56^dim ^cells (Figure 2A). The CD16^neg^/CD56^bright ^NK cells were either negative or presented very low levels of Siglec-9 (Figure 2A). A similar expression pattern for Siglec-9 was also observed on NK cells from PB and peritoneal fluid (PF) of epithelial ovarian cancer patients (Figure 2A).

As mentioned previously, sMUC16 is detected only on 30-40% of the CD16^pos^/CD56^dim ^NK cells isolated from the PB and PF of ovarian cancer patients, the same subset of NK cells that also are likely to express Siglec-9. We therefore performed multi-color flow cytometry experiments to determine if sMUC16 was selectively binding to the Siglec-9^pos^/CD16^pos^/CD56^dim ^NK cell subset. While Siglec-9 was detected on the CD16^pos^/CD56^dim ^NK cells isolated from the PB of healthy donors, the level of sMUC16 on these cells was either undetectable or very low (Figure 2B, C). To the contrary, between 74-82% of the Siglec-9^pos^/CD16^pos^/CD56^dim ^NK cells isolated from the PB and PF of five ovarian cancer patients were also positive (double positive) for sMUC16 (Figure 2C). The differences between the percentage of patient PB- and PF-derived CD16^pos^/CD56^dim ^NK cells that were positive for Siglec-9 and sMUC16 were statistically not significant (Figure 2C).

### Siglec-9 expression on B cells and monocytes

The PB and PF derived mononuclear cells (PBMC and PFMC) contain B cells, T cells, and monocytes in addition to the NK cells. Only a very minor subset (less than 2%) of the PBMC and PFMC derived CD3^pos ^T cells expressed Siglec-9 (data not shown). Furthermore, only low levels of Siglec-9 was observed on these T cells. We generally did not observe sMUC16 binding to the small percentage of these Siglec-9^pos ^T cells. However, because of the low number of events of Siglec-9^pos ^T cells and low levels of expression of this receptor, we were unable to obtain statistically relevant information on sMUC16 binding to T cells of all of the healthy donors and ovarian cancer patients recruited in this study. Hence, T cells were not further analyzed for sMUC16 binding and Siglec-9 expression.

**Figure 3:**
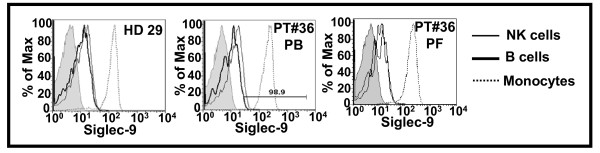
**Expression of Siglec-9 on NK cells, B cells and monocytes**. PBMC from healthy donors (HD) and PBMC and PFMC from patients (PT) were labeled with fluorophore conjugated anti-CD3, -CD19, -CD16, -CD33, -CD45, -CD56, and -Siglec-9 antibodies. Cells were analyzed by flow cytometry. Live and single events were gated and expression of Siglec-9 on CD16^pos^/CD56^dim ^(NK) cells, CD19^pos ^B cells, and CD33^pos ^monocytes was determined. Data shown for HD #29 and PT#36 is representative of that obtained for four healthy donors and five ovarian cancer patients, respectively.

Approximately 20% of CD19^pos ^B cells and greater than 90% of the CD33^pos ^monocytes were Siglec-9^pos ^(Figure 3). The expression level of Siglec-9 on B cells and NK cells was comparable. However, the entire population of the CD33^pos ^monocytes expressed several fold higher levels of Siglec-9 as compared to the B cells and NK cells (Figure 3).

### sMUC16 is present on Siglec-9^pos ^B cells and monocytes

**Figure 4:**
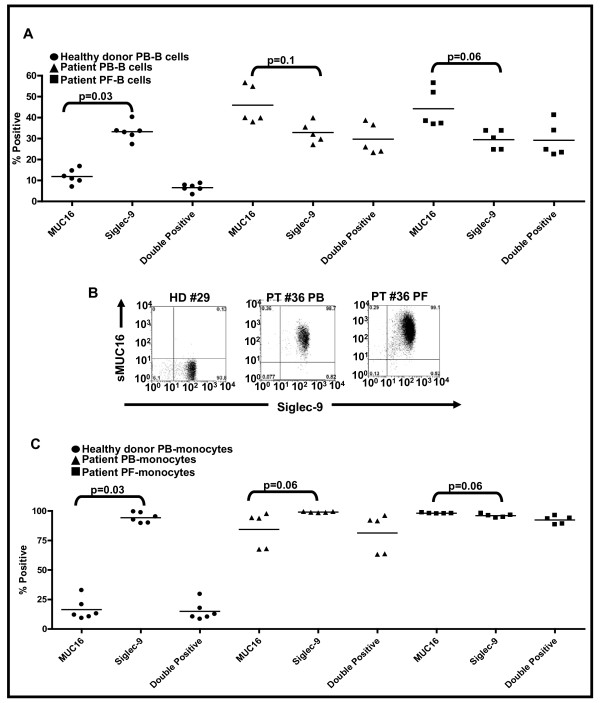
**sMUC16 predominantly binds to Siglec-9^pos ^B cells and monocytes**. B cells (CD19^pos^) and monocytes (CD33^pos^) present in the PBMC of healthy donors (HD) and PBMC and PFMC of ovarian cancer patients (PT) were identified by flow cytometry. ***A***, Siglec-9 expression and sMUC16 binding on B cells was determined and double positive events were gated. Data shown is mean of percent positive events for six HD and five PT PBMC samples. ***B***, High level of expression of Siglec-9 and a correspondingly high level of sMUC16 binding was observed on monocytes. Data shown is for HD #29 and PT #36 and is representative of the cumulative data, ***C***, obtained for six HD and five PT.

If sMUC16 was binding to NK cells via Siglec-9, this mucin should also be present on the Siglec-9^pos ^B cells and monocytes. This was indeed the case since 65-70% of the total sMUC16^pos ^B cells were also Siglec-9^pos ^(Figure 4A). In the case of monocytes the correlation between sMUC16 binding and Siglec-9 expression was even more striking. Greater than 95% of the sMUC16^pos ^monocytes were Siglec-9^pos ^and vice versa (Figure 4B and 4C). Overall, the mean fluorescent intensity for sMUC16 binding to the B cells, NK cells, and monocytes matched the expression levels of Siglec-9 on these immune cell types (data not shown).

### sMUC16 binds to Siglec-9

**Figure 5:**
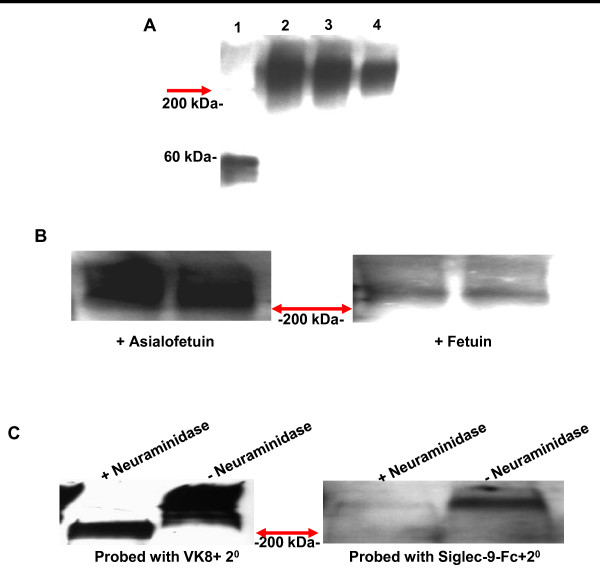
**Siglec-9 binds to sialylated glycans of sMUC16**. ***A***, Fetuin (lane 1) or sMUC16 (300 U of CA125, lane 2; 200 U of CA125, lane 3; and 100 U of CA125, lane 4) were separated by SDS-PAGE, transferred to a PVDF membrane and overlaid with Siglec-9-human Fc chimera (2.5 nM). Binding of Siglec-9-human-Fc was detected using horseradish-peroxidase-labeled mouse anti-human-Fc secondary antibody. ***B***, sMUC16 (250 U of CA125/lane) purified from the spent media of OVCAR-3 cells was loaded in duplicate on both blots. Siglec-9-human-Fc chimera was overlaid on the membrane in the presence of 7.5 nM asialofetuin (left panel) or fetuin (right panel). ***C***, sMUC16 was desialylated with neuraminidase from *Clostridium perfringens*. Binding of neuraminidase treated and untreated sMUC16 samples (250 U of CA125/lane) to VK-8 (left panel) or Siglec-9-human-Fc (right panel) was detected by Western blotting. Red arrows in all panels indicate interface between the stacking and separating gels. sMUC16 is typically retained in the stacking gel under the electrophoresis conditions used to develop these western blots. Migration of the 200 kDa (***A-C***) and 60 kDa (***A***) molecular weight markers is shown.

In Western blot analysis sMUC16 purified from OVCAR-3 cells was detected by the Siglec-9-human Fc chimera (Figure 5A). Fetuin, an α2,3-sialic acid expressing glycoprotein, blocked the binding of Siglec-9 human Fc chimera to sMUC16 (Figure 5B). Asialofetuin under identical conditions did not abrogate the binding of Siglec-9-human Fc to sMUC16 (Figure 5B).

To further demonstrate that Siglec-9-human Fc was recognizing the sialic acids of sMUC16 the mucin was digested with neuraminidase. Efficient desialylation of sMUC16 was demonstrated by the increased migration of asialo-sMUC16 detected by Western blot analysis using the anti-MUC16 antibody VK-8 (Figure 5C). Enzymatically desialylated sMUC16 was not recognized by the Siglec-9-human Fc chimera (Figure 5C).

**Figure 6:**
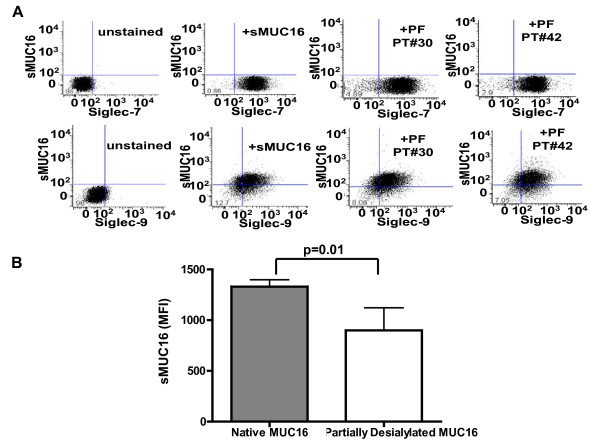
**sMUC16-Siglec-9 binding specificity**. ***A***, Siglec-7 (top panel) and Siglec-9 (bottom panel) expressing Jurkat cells were incubated for 24 h in media, media containing OVCAR-3 derived sMUC16 (50,000 U of CA125/ml), or in 10% media containing 90% peritoneal fluid (PF) from ovarian cancer patients (PT #30 and PT #42). The VK-8 antibody was used to detect binding of sMUC16 to the cells by flow cytometry. ***B***, Siglec-9 expressing Jurkat cells were incubated with native and partially desialylated sMUC16 (50,000 U of CA125/ml) for 3 h. Cells were labeled with VK8 and the amount of sMUC16 present on the surface was determined by flow cytometry. Mean and standard deviation of three separate experiments (p < 0.05) is shown.

Specificity of sMUC16 binding to Siglec-9 was determined using Jurkat cells transfected with cDNA coding for this I-type lectin [34]. The Siglec-9 expressing Jurkat cells were incubated with either sMUC16 (50,000 U of CA125/ml) purified from the conditioned media of OVCAR-3 cells or with peritoneal fluid from two ovarian cancer patients. After culture for 24 h (Figure 6A), sMUC16 was detected on Siglec-9 expressing Jurkat cells. Matched Jurkat cells that expressed Siglec-7 instead of Siglec-9 did not show any binding of sMUC16 under identical conditions (Figure 6A).

To further demonstrate that sMUC16 was binding to Siglec-9 expressing Jurkat cells via its sialic acid residues, purified mucin was desialylated with neuraminidase from *Clostridium perfringens*. The limited availability of purified sMUC16 compelled us to conduct neuraminidase treatment in cell culture compatible buffer (pH 7.4). The neuraminidase has an optimum pH of 5 [35]. Hence, digestion with this neuraminidase at pH 7.4 yielded only partial desialylation of sMUC16- as confirmed by western blotting experiments with VK-8 and biotinylated *Mackia amurensis *agglutinin (data not shown). The partially desialylated sMUC16 exhibited a 20-50% reduction in the binding to Siglec-9 expressing Jurkat cells (Figure 6B).

### Siglec-9 promotes immune cell-tumor cell adhesion via csMUC16

MUC1 binding to Siglec-4a (myelin associated glycoprotein) contributes to perineural adhesion of pancreatic cancer cells [36]. We therefore investigated if Siglec-9 could also promote immune cell-tumor cell adhesion via csMUC16 expressed on ovarian cancer cells.

To demonstrate the ability of Siglec-9 to act as a cell binding receptor, we utilized MUC16 knock down OVCAR-3 subline csMUC16^neg^OVC and MUC16 expressing OVCAR-3 subline MUC16^pos^OVC [16,37]. The csMUC16^neg^OVC cells express an endoplasmic reticulum localized scFv fragment of the VK8 antibody that prevents expression of csMUC16 and sMUC16. The OVCAR-3 subline csMUC16^pos^OVC expresses endoplasmic reticulum localized scFv fragment of an irrelevant mouse antibody that does not interfere with csMUC16 and sMUC16 expression.

**Figure 7:**
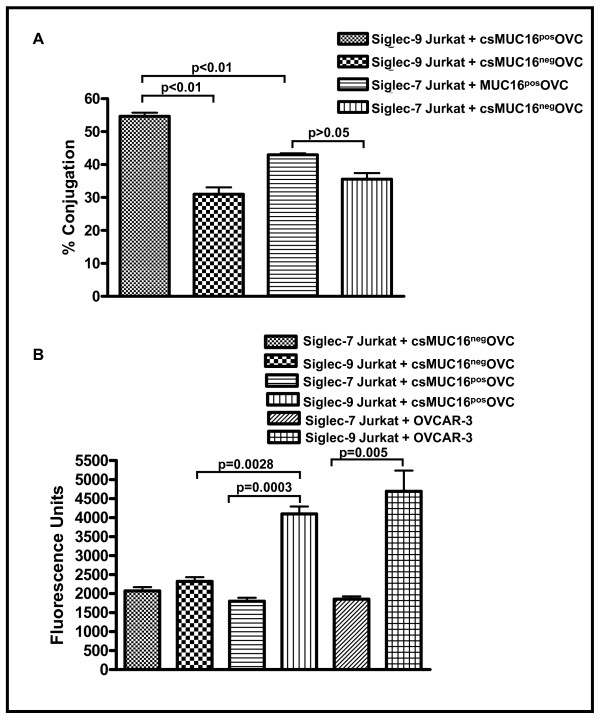
**Siglec-9 binds to csMUC16 and mediates immune cell-target cell binding**. ***A***, Siglec-9 expressing Jurkat cells were stained with CellTracker Blue and MUC16^neg^OVC or MUC16^pos^OVC cells were stained with CellTracker Green. The Jurkat cells and tumor targets were mixed in 1:1 ratio, incubated for 30 min at room temperature and conjugate formation was determined by flow cytometry as described previously [16]. Mean and standard deviation data for percent conjugation obtained from three independent flow cytometry experiments are summarized in the bar chart. ***B***, A direct 96-well plate-based cell adhesion assay was also used to measure binding of MUC16^pos^OVC and Siglec-9 expressing Jurkat cells. Ovarian tumor cells were plated in 96-well plates. Calcein AM labeled Siglec-9^pos ^or Siglec-7^pos ^Jurkat cells were added to the wells. After 25 min incubation wells were washed three times and the amount of fluorescence remaining in the wells was measured. Data shown is mean of six experiments.

Using two separate cell adhesion assays it was determined that Siglec-9 expressing Jurkat cells formed approximately 1.5-2-fold more cell conjugates with csMUC16^pos^OVC cells as compared to csMUC16^neg^OVC cells. Siglec-7 expressing Jurkat cells, most likely via Siglec-7-independent mechanisms, did indeed form conjugates with both csMUC16^neg^OVC and csMUC16^pos^OVC cells (Figure 7A, B). However, significantly more conjugate formation was observed between Siglec-9 expressing Jurkat cells and csMUC16^pos^OVC cells than between Siglec-7 expressing Jurkat and csMUC16^pos^OVC cells (Figure 7A, B).

**Figure 8:**
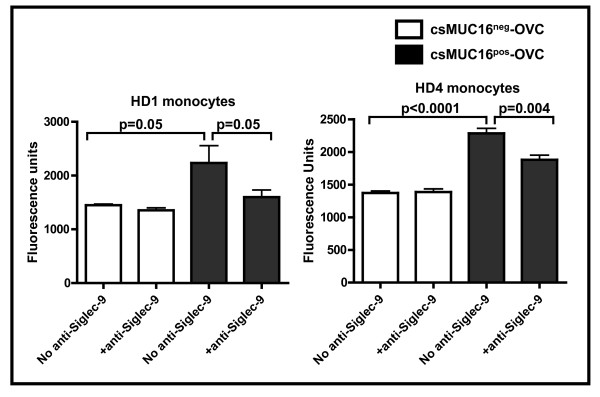
**Monocytes bind to tumor cells via csMUC16-Siglec-9 interaction**. Monocytes were purified from PBMC of healthy donors using the RosetteSep monocyte isolation kit (Stem Cell Technologies). The monocytes were labeled with Calcein AM and added to wells containing either csMUC16^pos^-OVC or csMUC16^neg^-OVC. After incubation and washing, the Calcein-labeled monocytes bound to the tumor cells were measured on a fluorescence plate reader. To demonstrate that binding was occurring via Siglec-9, matching Calcein AM-labeled monocytes were pre-treated with anti-Siglec-9 blocking antibody (Clone 191240, R&D Systems) prior to conducting the cell binding assay. Data shown is mean of three independent measurements.

Cell binding assays were also performed with monocytes isolated from the PB of healthy donors. Similar to results obtained with the Siglec-9 and Siglec-7 expressing Jurkat cells, basal level of binding of monocytes was observed with the csMUC16^neg^-OVC. However, in five independent experiments conducted with monocytes isolated from three healthy donors, increased binding of the monocytes to the csMUC16^pos^-OVC as compared to the csMUC16^neg^-OVC was consistently observed. The increased binding of monocytes to the csMUC16^pos^-OVC was significantly inhibited by an anti-Siglec-9 blocking antibody (Figure 8) [38].

## Discussion

Mucins are bulky, heavily glycosylated molecules that are known to function in diverse biological scenarios [39,40]. One of the intriguing properties of mucins is their ability to act as both adhesive as well as anti-adhesive molecules. The bulky nature of the mucins coupled with the presence of negatively charged oligosaccharides contributes to their anti-adhesive properties. On the other hand, specific oligosaccharide and peptide sequences of mucins may serve as ligands for cell adhesion receptors such as the selectins and integrins thereby allowing mucins to act as pro-adhesion molecules.

Similar to other mucins, MUC16 also exhibits both anti- and pro-adhesive properties. By serving as a ligand of mesothelin, MUC16 mediates binding between ovarian cancer cells and the mesothelium [15,16]. The anti-adhesive property of MUC16 has been clearly demonstrated in the human endometrial tissues [3]. Our recent work [19] shows that the anti-adhesive property of MUC16 allows the tumor cells to circumvent immune synapse formation with cytotoxic NK cells.

In the current study, we have now identified a new mechanism by which csMUC16 may increase the contacts between ovarian cancer cells and immune cells. Here, we have identified Siglec-9 as the immune cell receptor for sMUC16 and csMUC16. The binding of csMUC16 to Siglec-9 allows immune cells to form direct contact with the ovarian cancer cells. Thus, while on the one hand, csMUC16 blocks immune synapse formation, the results of the current study indicate that the mucin may allow increased immune cell binding to ovarian cancer cells. These seemingly contradictory observations can, however, be reconciled.

Siglec-9 is an inhibitory receptor that signals via its proximal Immunoreceptor Tyrosine-based Inhibition Motif (ITIM). Ligand binding results in the phosphorylation of tyrosine residues in the ITIM and the recruitment and activation of the phosphatases SHP-1 and SHP-2 [34,41]. Because of the activation of these two phosphatases, binding of Siglec-9 to its ligands results in potent inhibition of NK cell and T cell anti-tumor functions [34,41]. Furthermore, engagement of Siglec-9 on monocytic cells results in the secretion of the immunosuppressive cytokine IL-10 [42].

In over 20 ovarian cancer patient samples analyzed to date we have invariably found sMUC16 attached to the surface of specific immune cell subsets [20]. Data presented in the current study demonstrates that sMUC16 predominantly binds to NK cells, B cells and monocytes via Siglec-9. These observations suggest that sMUC16-Siglec-9 binding may trigger an inhibitory response in circulating cytotoxic immune cells even before they interact with the tumor. In other words, sMUC16-Siglec-9 binding may result in inhibitory priming of the immune cells leading to compromised anti-tumor immunity. The occurrence of immune suppression in ovarian cancer patients is a common observation [43-50]. sMUC16-Siglec-9 binding is likely one of many molecular events that contribute to attenuation of anti-tumor immune responses. Our ongoing work is focused on determining the cytokine release and activation capacity of immune cells once sMUC16 binds to Siglec-9.

Competent Siglec-9^pos ^immune cells entering the tumor microenvironment may adhere to the cancer cells via csMUC16. Siglecs have previously been shown to act as adhesion receptors. MUC1, another prominent member of the mucin family has been previously shown to be a ligand for Siglec-1 and Siglec-4a [36,51]. MUC1-Siglec-4a binding promotes the perineural invasion of pancreatic cancer cells [36]. Because Siglec-9 is an inhibitory receptor, the interaction between csMUC16 and Siglec-9 may not necessarily lead to enhanced immune recognition of the tumor but instead cause protection of the cancer cells.

Finally, the anti-adhesive properties of csMUC16 allow the cancer cells to evade immune recognition by the Siglec-9^neg ^immune cells. As shown in our other study [19], csMUC16 prevents efficient immune synapse formation between cancer cells and NK cells. Thus, the pro-adhesive and anti-adhesive properties of csMUC16 are of great benefit to the tumor and work in tandem to provide immune protection during the development and progression of ovarian cancer.

The identification of Siglec-9 as the receptor for MUC16 also has significance in the development of a novel test to monitor ovarian cancer progression. Serum CA125 levels are routinely monitored in patients who are already undergoing treatment for ovarian cancer. A decrease in serum CA125 indicates a positive response to the treatment regimen, whereas increase in this marker above a nadir concentration suggests recurrence of the tumor [13,14,52]. One major factor that impairs efficient detection of CA125 (MUC16) and other mucinous cancer antigens in the sera is their degradation by the liver. Circulating mucins are rapidly cleared by hepatic reticuloendothelial cells via lectins specific for galactose/N-acetylgalactosamine (tolerating α2,3-sialylation), mannose/N-acetylgalactosamine-4-sulfate, and hyaluronan receptors and the scavenger receptors [53]. Hepatic uptake of mucins therefore hampers efficient detection of these tumor antigens in cancer patients. The binding of sMUC16 to Siglec-9 on the surface of specific immune subsets may preclude the uptake and degradation of the mucin. Therefore, immune cell bound sMUC16 may prove to be a more reliable source for measuring the changes in CA125 levels in ovarian cancer patients. We are currently investigating the potential of monitoring immune cell bound sMUC16 as a more sensitive diagnostic assay to predict recurrence of ovarian cancer.

## Conclusions

In this study we have identified Siglec-9 as the immune cell receptor for MUC16. Our previous studies have indicated that sMUC16 and csMUC16 serve an important immunomodulatory role, allowing tumor cells to escape recognition by NK cells. Siglec-9 is an inhibitory receptor. The demonstration that sMUC16 and csMUC16 are ligands of Siglec-9 now allows us to further define the molecular mechanisms leading to the attenuation of immune responses by this mucin.

## Methods

### Sample Processing

The peripheral blood and peritoneal fluid samples were obtained from nine epithelial ovarian cancer patients (designated PT#24, PT#36, etc) recruited at the time of their initial diagnosis. All patients and healthy donors (designated HD#1, HD#2, etc) signed an informed consent, and the studies were approved by the Institutional Review Board of University of Wisconsin-Madison. The peripheral blood and peritoneal fluid samples were processed as described previously [20]. The serum CA125 levels of patients and healthy donors and other demographic information is provided as Supplementary Table 1.

### Flow Cytometry

Cryopreserved or freshly obtained mononuclear cells from the peripheral blood or peritoneal fluid samples were analyzed. The cells were processed and labeled with antibodies as described previously [20]. For blocking purposes, cells were first incubated for 15 minutes with goat antibody before staining with the anti-MUC16 antibody VK8, or the anti-galectin-1 antibody, LGALS-1 (Abnova, Taipei, Taiwan). After washing, FITC- or APC-conjugated goat anti-mouse (GAM) secondary antibody was added at 1:100 or 1:150 dilutions, respectively and the cells were incubated with mouse IgG for 15 minutes to block any additional Fab sites on the GAM secondary. The cells were then incubated with various cocktails of directly conjugated antibodies to stain for CD3 (APC-Cy7), CD45 (PerCP-Cy5.5), CD33 (PerCP-Cy5.5), CD56 (Alexa^® ^700), CD16 (PE-Cy7), CD19 (PE), Siglec-9 (FITC), or Siglec-7 (PE). After a final wash, cells were resuspended in 300 μL of phosphate buffered saline containing 1% bovine serum albumin (PBS-BSA). Immediately before data acquisition on an LSRII (Beckton Dickinson) flow cytometer, the viability indicator DAPI (1:300; BD Biosciences) was added to each sample. Each fluorophore was compensated by automatic computerized compensation. To compare the values of samples analyzed on different days, SPHERO™ Rainbow Fluorescent Particles (BD Biosciences) were used to set instrument voltages. Data was analyzed by FlowJo software (v. 8.5.3, TreeStar) and data plotting and statistical analysis was done using GraphPad Prism software (v. 4, GraphPad Software, Inc.).

### Western and Far-Western Blotting

The sMUC16 (isolated from culture media of OVCAR-3 cells as described earlier [10]) and the bovine glycoprotein, fetuin, were separated on a 4% stacking/7.5% resolving gel. The proteins were transferred to PVDF membrane, blocked with BSA and overlaid with either the VK-8 antibody or Siglec-9-human Fc chimera (2.5 nM) (R&D Systems). Horseradish peroxidase conjugated goat anti-mouse or a goat anti-human Fc secondary antibodies were used for detection.

For inhibition of Siglec-9 binding to sMUC16 by fetuin or asialofetuin, the Siglec-9-human Fc was pre-incubated for 5 min with 3-fold molar excess of fetuin or asialofetuin. The Siglec-9-human-Fc along with the fetuin or asialofetuin was overlaid on membranes blotted with sMUC16 and binding was detected as described above.

### Desialylation of MUC16

sMUC16 (2000 U of CA125) isolated from the conditioned media of OVCAR-3 cells was treated with 1U of neuraminidase from *Clostridium perfringens *(Sigma Aldrich) in phosphate buffered saline (pH 7.4) in a total of 100 μL for 16 h at 37°C. Control sMUC16 samples were treated under identical conditions except that neuraminidase was not added.

For desialylation experiments of immune cells, PBMC (1-2 × 10^6^) freshly isolated from ovarian cancer patients were resuspended in 500 μl phosphate buffered saline (pH 7.4). Neuraminidase (0.5 U/ml) was added and the cells were incubated at 37°C for 15 min. For controls, PBMC's from the same patients were incubated under identical conditions in buffer but neuraminidase was not added. After digestion, the test and control cells were washed with phosphate buffered saline containing 1% bovine serum albumin and sMUC16 bound to the cells was determined by flow cytometry.

### Binding of sMUC16 to Siglec expressing Jurkat cells

The Siglec-7^pos ^and Siglec-9^pos ^Jurkat cells were developed as described in a previous report [34]. The cells were washed in culture media (RPMI 1640 containing 10% fetal calf serum, non-essential amino acids and G418) and incubated for different time points in media containing native or desialylated sMUC16 (25,000-50,000 U CA125/ml) isolated from OVCAR-3 cells, or in a mixture of 90% peritoneal fluid, and 10% fetal calf serum. Following incubation, the cells were washed twice and stained for flow cytometry as described above.

### Cell conjugation assays

MUC16 knockdown OVCAR-3 (MUC16^neg^OVC, previously designated as #7 cells) and the corresponding MUC16 expressing OVCAR-3 (MUC16^pos^OVC, previously designated as #12 cells) sublines were obtained as reported previously [16,37]. Siglec-9 and Siglec-7-expressing Jurkat cells were labeled with 5 μM CellTracker Blue (Invitrogen) for 25 minutes at 37°C in 5% CO_2_. Concurrently, MUC16^neg^OVC and MUC16^pos^OVC were harvested and dyed with 1.25 pM CellTracker Green (Invitrogen) under identical conditions. After washing off excess dye with PBS, cells were resuspended in PBS containing 1% BSA. Siglec-9 and Siglec-7 expressing Jurkat cells were placed in flow tubes with either MUC16^neg^OVC or MUC16^pos^OVC cells in a 1:1 ratio and centrifuged for 2 minutes at 100 g. Tubes were incubated for 30 minutes at room temperature, and vortexing was avoided. Immediately before data acquisition on an LSRII (Beckton Dickinson) flow cytometer, the viability indicator DAPI (BD Biosciences) was added to each sample.

For microtiter plate based assays, the MUC16^pos^-OVC, MUC16^neg^-OVC and OVCAR-3 cells (1 × 10^5^/well) were cultured for two days in 96-well plates. On the day of the assay, Siglec-9 and Siglec-7 expressing Jurkat cells or monocytes isolated from the blood of healthy donors were labeled with Calcein AM (0.1 μM) for 30 mins at 37°C. Calcein AM labeled Jurkat cells or monocytes (1 × 10^5^/well) were added to wells containing the ovarian tumor targets. Monocytes were freshly isolated using the RosetteSep monocyte isolation kits (Stem Cell Technologies). Tissue culture plates were incubated for 25 min at 37°C in a 5% CO_2 _environment. After incubation, wells were washed 4-6 times with PBS containing 1% BSA. The plates were analyzed on a fluorescent plate reader to determine the binding of Siglec-9 and Siglec-7 expressing Jurkat cells to the ovarian tumor targets.

## Competing interests

JAB, JC, and MSP are co-inventors on a United States patent application filed through the Wisconsin Alumni Research Foundation (WARF) on the use of immune cell bound sMUC16 as a diagnostic assay. As co-inventors, JAB, JC, and MSP have received research funding and minor compensation from WARF. None of the other authors have any financial conflict of interest.

## Authors' contributions

JAB conducted the majority of the research SH, JAAG, SP, and AK conducted some of the experiments and provided technical support. SA and HJG provided reagents for galectin-1 analysis and helped in designing galectin expression experiments. JPC provided the Siglec-9 and Siglec-7 reagents and provided intellectual input for this manuscript. JC provided technical and clinical expertise for this project. MSP is the corresponding author and helped in study design, data interpretation and preparation of the manuscript. All authors have read and approved the final version of this manuscript.

## Supplementary Material

Additional file 1 **Galectin-1 expression on NK cell subsets**. The peripheral blood mononuclear cells were isolated from healthy donors. The cells were stained with a panel of fluorophore conjugated anti-CD3, CD16, CD45, CD56, and unconjugated galectin-1 antibodies. Binding of antibody to surface galectin-1 was detected by FITC-labeled goat anti-mouse secondary antibody. Cells were analyzed by flow cytometry. Live, single events were gated and galectin expression on the CD16^pos^/CD56^dim ^and CD16^neg^/CD56^bright ^NK cells was determined. Data shown is for NK cells from HD#25 and is representative of results obtained from three healthy donors. Click here for file
