# Supplementary figures and images for: Identification of Siglec-9 as the receptor for MUC16 on human NK cells, B cells, and monocytes
Source: Mol Cancer. 2010 May 24;9:118. doi: 10.1186/1476-4598-9-118 (PMC2890604; doi:10.1186/1476-4598-9-118)

## Slide 1
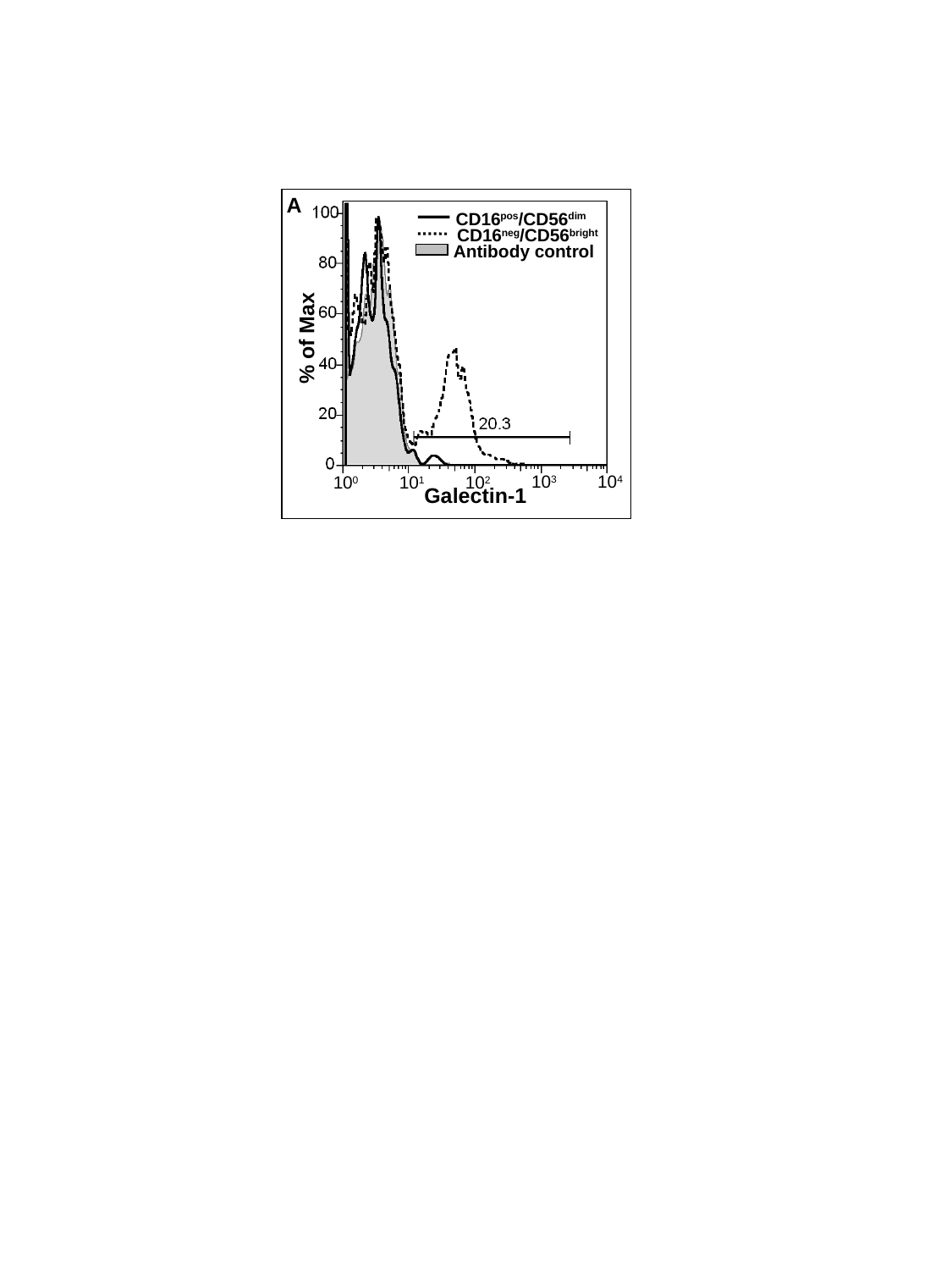

A
CD16pos/CD56dim
CD16neg/CD56bright
Antibody control
% of Max
103
104
101
102
100
Galectin-1

Supplement: Additional file 1 — Galectin-1 expression on NK cell subsets. The peripheral blood mononuclear cells were isolated from healthy donors. The cells were stained with a panel of fluorophore conjugated anti-CD3, CD16, CD45, CD56, and unconjugated galectin-1 antibodies. Binding of antibody to surface galectin-1 was detected by FITC-labeled goat anti-mouse secondary antibody. Cells were analyzed by flow cytometry. Live, single events were gated and galectin expression on the CD16pos/CD56dim and CD16neg/CD56bright NK cells was determined. Data shown is for NK cells from HD#25 and is representative of results obtained from three healthy donors. [file 1476-4598-9-118-S1.PPT]
